# Contextualizing HIV testing experiences within the HIV prevention cascade: qualitative insights from refugee youth in Bidi Bidi refugee settlement, Uganda

**DOI:** 10.1186/s12889-024-20135-2

**Published:** 2024-09-27

**Authors:** Carmen Helen Logie, Moses Okumu, Miranda Loutet, Madelaine Coelho, Alyssa McAlpine, Frannie MacKenzie, Simon Odong Lukone, Nelson Kisubi, Hakim Kalungi, Okello Jimmy Lukone, Peter Kyambadde

**Affiliations:** 1 Factor-Inwentash Faculty of Social Work, University of Toronto, 246 Bloor Street West, Toronto, ON M5S 1V4 Canada; 2 Women’s College Research Institute, Women’s College Hospital, Toronto, Canada; 3 United Nations University Institute for Water, Environment, and Health, Hamilton, Canada; 4 Centre for Gender & Sexual Health Equity, Vancouver, Canada; 5 School of Social Work, University of Illinois at Urbana Champaign, Urbana, USA; 6 School of Social Sciences, Uganda Christian University, Mukono, Uganda; 7 Dalla Lana School of Public Health, University of Toronto, Toronto, Canada; 8 Department of Sociology, University of Toronto, Toronto, Canada; 9 Uganda Refugee and Disaster Management Council (URDMC), Arua, Uganda; 10 International Rescue Committee, Yumbe, Uganda; 11 National AIDS Coordinating Program, Ugandan Ministry of Health, Kampala, Uganda; 12 Most at Risk Population Initiative (MARPI), Kampala, Uganda

**Keywords:** Refugee and internally displaced, HIV testing, HIV self-testing, Humanitarian, Uganda, Adolescent and youth, HIV prevention

## Abstract

**Background:**

There remain key knowledge gaps regarding HIV testing needs and priorities among refugee youth in low and middle-income country (LMIC) humanitarian settings. The HIV prevention cascade framework focuses on three domains (motivation, access, effective use) central to prevention uptake, yet is understudied in relationship to HIV testing, particularly among refugee youth. Uganda is an exemplar context to explore refugee youth HIV testing needs and priorities as it hosts 1.5 million refugees and is Africa’s largest refugee hosting nation. In this study, we explored perceptions and experiences regarding HIV testing among refugee youth living in Bidi Bidi refugee settlement, Uganda.

**Methods:**

We conducted a community-based research study in Bidi Bidi Refugee Settlement, one of the world’s largest refugee settlements with over 195,000 residents. This qualitative study involved four focus groups (2 with young women, 2 with young men) with refugee youth aged 16–24 living in Bidi Bidi refugee settlement. We applied thematic analysis informed by the HIV prevention cascade to understand domains of motivation, access, and effective use that emerged as salient for HIV testing engagement.

**Results:**

Participants (*n* = 40; mean age: 20 years, standard deviation: 2.2) included refugee young women (*n* = 20) and young men (*n* = 20), of whom 88% had a lifetime HIV test and 58% had ever heard of HIV self-testing. Participant discussions described HIV testing motivation was influenced by dimensions of: HIV treatment and testing knowledge; risk perception; positive and negative consequences of use; and social norms regarding gender and age. Access to HIV testing was shaped by: limited availability; distance and language barriers; confidentiality concerns; and affordability. Effective use of and engagement with HIV testing was related to HIV serostatus knowledge self-efficacy and in/equitable partner dynamics.

**Conclusions:**

Complex, multi-level factors shape motivation for, access to, and effective use of HIV testing among refugee youth in Bidi Bidi. Findings align with the HIV prevention cascade framework that helps to identify gaps to inform intervention development with youth in humanitarian settings. HIV testing approaches tailored for refugee youth in contexts such as Bidi Bidi can foster HIV prevention and treatment literacy, gender equity, gender-based violence prevention, and intersectional stigma reduction.

**Supplementary Information:**

The online version contains supplementary material available at 10.1186/s12889-024-20135-2.

## Introduction

Key knowledge gaps remain regarding HIV testing priorities among refugee youth in low and middle-income country (LMIC) humanitarian settings [1]—where most of the world’s 103 million forcibly displaced persons live [2]. Indeed, a recent commentary declared that *“displaced populations are being neglected in efforts to end the AIDS epidemic”* (p. 5) [1]. Uganda is an exemplar context to explore refugee youth HIV testing as it hosts 1.5 million refugees [2]. Most refugees (92%) in Uganda live in refugee settlements, and Bidi Bidi is the second largest refugee settlement with over 195,000 residents—one-quarter youth aged 15–24 [3]. Prior research documents unmet HIV testing needs among refugee youth in Uganda, estimated at 43.5% among urban refugee youth in Kampala [4]. This falls short of the UNAIDS goal of 95% of people knowing their status to achieve an AIDS Free Generation by 2030 [5].

This reflects the situation among non-refugee youth in Uganda, where HIV testing is suboptimal among general populations of youth at large aged 15–19 (54% of young women ever tested, and 44% of young men) [6]. HIV prevention knowledge is also low (45.4%) among general populations of youth aged 15–24 [7]. Low HIV testing and knowledge are concerning as HIV prevalence in Uganda at large is estimated at 5.5% among adults (2.5% among young women and 1.0% among young men) [7]. HIV testing among refugee youth living in rural refugee settlements is particularly important to understand as humanitarian settings in low and middle-income contexts may experience constrained access to health clinics and youth-friendly HIV services [8]. HIV testing, a key entry point into accessing HIV treatment and prevention services, is central to the HIV prevention cascade [9, 10] yet remains understudied with refugee adolescents and youth in Uganda [1].

The HIV prevention cascade integrates epidemiological, behavioural, and social perspectives to examine factors that shape HIV prevention engagement [9, 11]. Applying the HIV prevention cascade framework can inform intervention development through its focus on three domains relevant to prevention uptake: *motivation* (e.g., social norms), *access* (e.g., availability), and *effective use* (e.g., self-efficacy) [12]. HIV vulnerability among refugees is shaped by a complex interplay of structural, social, and behavioural factors experienced pre-migration, in transit, and post-migration [1, 8]. The HIV prevention cascade framework offers a germane approach to explore HIV testing gaps with refugee youth in LMIC.

HIV testing barriers may be exacerbated among refugee youth due to intersecting stigma (e.g., refugee-related, HIV-related) [13], logistic barriers [14], and inequitable relationship dynamics [15]. HIV testing among refugee youth in rural settlements is underexplored, yet these contexts may experience constrained access to health clinics and youth-friendly HIV services [8].

The *motivation*, *access*, and *effective use* domains of the HIV prevention cascade [12] are a promising framework to understand HIV testing with refugees in Uganda. Within the *motivation* domain, interest to participate in HIV prevention is related to knowledge, risk perception, consequences of use, and social norms [16]. To illustrate, decreased HIV testing motivation among refugee youth in Kampala was associated with fear of testing HIV positive, low risk perception due to beliefs that HIV is a “Ugandan” disease, and stigma toward sexually active youth [13]. A study with refugees in Uganda’s Nakivale refugee settlement reported that insufficiently met basic needs (i.e., food, shelter) decreased motivation for HIV testing [17].

The second domain, *access*, includes availability, ease of access, acceptability of provision, and affordability [16]. Refugees in humanitarian settlements may experience HIV testing barriers, including far distances to clinics [18], supply stockout, and long clinic waiting times [8, 19, 20]. Additionally, healthcare confidentiality concerns, low literacy, and language-related difficulties presented HIV testing barriers among urban refugee youth in Kampala [13].

The *effective use* domain includes skills, self-efficacy, and partner dynamics [16]. Among non-refugee youth, accessing social support during HIV testing processes is a skill that can influence HIV prevention and treatment engagement [21, 22]. Similarly, among refugees in Nakivale, social support was associated with increased likelihood of linkage to HIV care following an HIV-positive diagnosis [23]. Urban refugee youth in Kampala expressed hesitancy to engage in partner testing due to concerns of gender-based violence and potential relationship breakdown [13].

HIV self-testing (HIV-ST) is a youth-friendly strategy to increase HIV testing uptake that can reduce access barriers compared with clinic-based HIV testing [24, 25], including in humanitarian settings [8, 13]. HIV-ST has the potential to reduce stigma exposure by allowing an individual to test in a private location [26, 27]. Moreover, HIV-ST allows for more flexible and convenient testing access which can reduce the logistic barriers (travel time and cost) associated with facility-based HIV testing [27]. Among youth in Nigeria for instance, autonomy, accessibility, and stigma reduction were listed as enablers of HIV-ST uptake [28].

To address knowledge gaps regarding optimizing youth HIV testing strategies in refugee settlements, we applied the HIV prevention cascade framework [16] to explore perceptions and experiences regarding HIV testing, including HIV-ST, among refugee youth aged 16–24 in Bidi Bidi refugee settlement, Uganda.

## Methods

We conducted a community-based research study in Bidi Bidi Refugee Settlement that involved collaboration between researchers, government agencies, and community-based organizations. The qualitative data reported here was collected in August 2021 to inform the design of a randomized control trial to test the effectiveness of providing HIV-ST and edutainment comics to increase refugee youth’s HIV testing uptake [29].

### Ethical approval

Ethical approval was obtained from Mildmay Uganda Research Ethics Committee (REF-0802-2021), Uganda National Council for Science and Technology (SS884ES), and the University of Toronto Research Ethics Board (37496). All participants provided written informed consent prior to participation.

### Study setting

The study was conducted in Bidi Bidi refugee settlement in Northern Uganda close to the South Sudan border, with over 195,000 residents [3]. Our study was conducted in Zone 3 and Zone 4 Annex, which hosts 40% of Bidi Bidi’s residents [30]. Most of Bidi Bidi is comprised of women and children (85%), approximately one-quarter (24%) are youth aged 15–24, the majority of residents (80.1%) have no formal paid occupation, and 99% are from South Sudan [30]. In Bidi Bidi, while health centres offer free HIV testing and comprehensive HIV care, they do not yet offer HIV-ST kits [31, 32].

### Participants

We conducted four focus groups with 10 participants per group: two with young women and two with young men. Inclusion criteria: refugees aged 16–24 years, living in Bidi Bidi, and able to speak English, Bari and/or Juba Arabic. Peer navigators (PN), refugees aged 20–30 living in Bidi Bidi, fluent in English as well as Bari and/or Juba Arabic, worked with community partners to recruit eligible youth participants using convenience sampling with a word-of-mouth recruitment strategy.

### Data collection and analysis

Two research assistants facilitated four focus groups; each group was approximately one hour in duration, groups were separated by gender (two for women and two for men), and each included 10 participants. The focus groups were supported by two PNs, who provided real-time translation as needed. All focus groups were audio-recorded, transcribed verbatim, and Bari and/or Juba Arabic was then translated to English. During the focus group, a trained interviewer collected socio-demographic data including age, country of origin, education, and employment status.

We used a semi-structured discussion guide to explore knowledge and experiences with current HIV testing opportunities in Bidi Bidi. The discussion guide was piloted in Bidi Bidi with research assistants and subsequently edited based on feedback to enhance relevance and clarity. Following this discussion of HIV testing in general, participants were then asked perspectives specifically on HIV-ST. Focus group facilitators gave participants a brief description of HIV self-test kits (i.e., testing without going to the clinic by swabbing the inside of the cheek and results available within 20 min), showed them a kit, and explained the instructions that came with the test kit. Facilitators emphasised that it was important to go to the clinic to confirm the result if the HIV self-test is positive. Participants were then asked to reflect on acceptability and feasibility of using a HIV self-test kit, including motivations and challenges and any perceived gender differences regarding kit usage.

We used Dedoose, a cloud-based and cross-platform application, for coding transcripts [33]. We applied thematic analysis to explore patterns of meaning in the data as this approach is theoretically flexible and integrates both deductive and inductive analyses [34, 35]. Deductive analyses were informed by Moorehouse et al.’s HIV prevention cascade dimensions of *motivation*, *access*, and *effective use* [16]. Both inductive and deductive analyses were based on responses to prompts from the discussion guide, which included: (1) What helps young people in your community decide to get an HIV test? (2) What are some challenges young people in your community face for testing (e.g., stigma, location, hours)? (3) How are people living with HIV treated in your community?

## Results

Participants (women: *n* = 20; men: *n* = 20) ranged from 16 to 24 years old (mean age = 20, standard deviation (SD): 2.2), and most reported a lifetime HIV test (88%) (Table 1). Over half (58%) were aware of HIV-ST.

**Table 1:** Sociodemographic characteristics of refugee youth focus group participants in Bidi Bidi Refugee Settlement, Uganda (*n* = 40)

Sociodemographic characteristics	*n*(%), mean (SD), or median (IQR)
Age in years (mean, SD)	20 (2.2)
Gender(n,%)	
Women	20 (50)
Men	20 (50)
Born outside of Uganda (n, %)	
Yes	33 (83)
No	7 (17)
Highest level of education* (n, %)	
Less than secondary school	16 (40)
Completed secondary school	21 (53)
Attended some college or above	3 (7)
Employment status (n, %)	
Student	34 (87)
Employed part-time or looking for work	5 (13)
Test for HIV in lifetime	
Yes	35 (88)
No	5 (12)
Time in years since last HIV test in years among those who have tested in their lifetime (median, IQR)	1 (0, 2)
Aware of HIV self-testing	
Yes	23 (58)
No	17 (42)

**Fig. 1:**
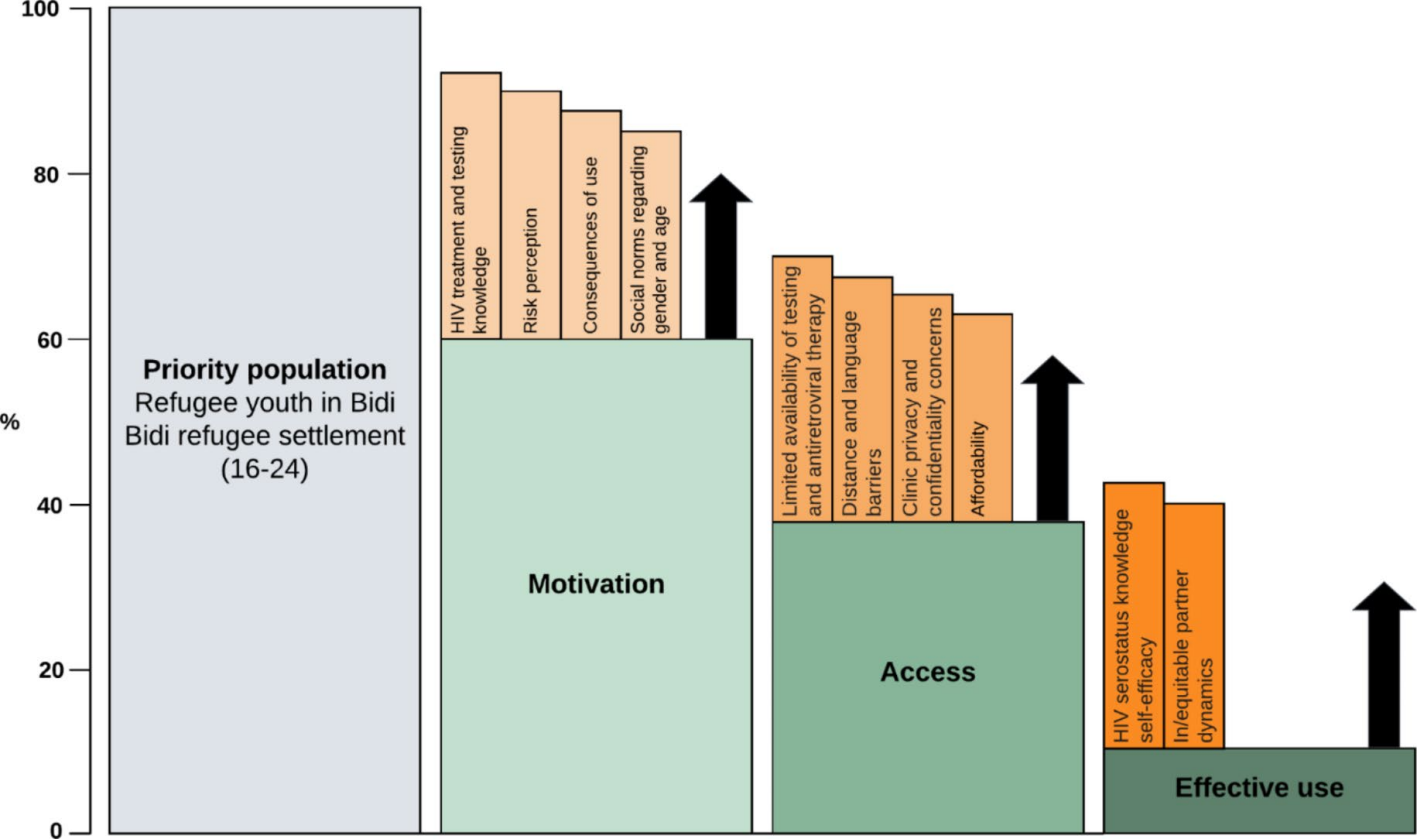
The HIV prevention cascade framework adapted from Moorhouse et al. [16] to understand HIV testing among refugee youth in Bidi Bidi refugee settlement, Uganda

We applied the HIV prevention cascade framework [16] to contextualize findings (Fig. 1). As illustrated in Fig. 1, participant discussions reveal HIV testing experiences and perspectives spanned *motivation*, *access* and *effective use* domains as detailed below.

### Motivation

Participant discussions indicate motivation for HIV testing is shaped by: HIV treatment and testing knowledge; risk perception; consequences of use; and social norms regarding gender and age.

#### HIV treatment and testing knowledge

Participant discussions highlighted varying levels of knowledge regarding HIV treatment, HIV testing, and how to use an HIV-ST kit. Participants with HIV testing and treatment knowledge (e.g., testing after exposure to HIV, post-exposure prophylaxis) seemed highly motivated to engage in HIV testing. Additionally, knowing about the effectiveness of antiretroviral therapy (ART) was a motivator for HIV testing. As a young man (age 23, focus group [FG] #2, identification number, [ID#] 3) explained: *“I go for check-up [HIV testing] because I want to get medication*,* as we all know HIV/AIDS has no cure*,* but the medication can help reduce the death rates. Because in the hospital if they find that you have HIV you can automatically get the medication.”*

Alternatively, low treatment literacy was associated with perceptions of HIV treatment as ineffective or nonexistent, and fear of dying from HIV which reduced testing motivation:

*Some people just fear because they know that HIV does not have cure*,* because if you go for HIV test and you find that you are positive there it will come automatically in your mind that you are going to die*,* so you start thinking within you like…I wasted my time going there for the test*,* I will not even get the medicine so better I relax at home and die. (man*,* age 19*,* FG#2*,* ID#2)*

Youth discussed knowledge barriers associated with the practical use of HIV-ST, including how to use or store HIV-ST kits. Others shared concerns about HIV-ST that reflect both low HIV treatment and testing literacy, such as not knowing what to do in the event of receiving a positive HIV-ST result. For example, a young woman accounted:

*To me it’s not good. Me*,* I don’t like this self-test because if you test yourself alone you can die in silence*,* you will not be able to tell your friends or call someone to advise you*,* you will die there alone. At least when you test at the health centre the doctor can know your status and can advise you accordingly. (woman*,* age 21*,* FG#3*,* ID#2)*

#### Risk perception

Participant testing motivation was impacted by the extent they viewed themselves as at risk for HIV. Youth discussed being motivated to test when not knowing one’s own or one’s sex partner’s HIV serostatus. To illustrate, a young woman explained:

*When you happen to have sex with someone*,* you think maybe you are not sure of him then… maybe people suspect him of having it*,* then you yourself start hearing*,* it will make you to at least go and test to prove whether you acquired it from him or not” (woman*,* age 20*,* FG#1*,* ID#9)*.

Participants who perceived themselves to be at risk for acquiring HIV appeared motivated to test and know their serostatus for their peace-of-mind. For example, one young man stated:

*For me the reason why I went for check-up is because I am still young as another colleague said. I still have interest in ladies*,* and I don’t know how many ladies I may have sex with in a month so this makes me go to the health centre to know if I am safe*,* so that if I am safe I can use protection and if I am not safe*,* I will need the doctor to give me advice. (man*,* age 18*,* FG#2*,* ID#1)*

Participants also noted low HIV acquisition risk perceptions, including not getting tested for HIV if a friend has tested HIV-negative. For example, one young woman (age 17, FG#1, ID#2) reported that: *“… when they go with friends*,* one friend gets tested: if the friend is [HIV]-negative then all of them say they are not going for testing again because since the friend is negative*,* they are also negative.”*

#### Consequences of use

Motivation to test was higher in discussions which emphasized positive consequences of HIV testing, such as knowing one’s status and feeling supported, and was lower with potential negative consequences such as shame, suicidality, and confidentiality breaches. Youth discussed knowing one’s own HIV status as a positive consequence of HIV testing. For example, one young man (age 20, FG#2, ID#8) reported: *“for me I take an HIV test so that I can find out whether I have the virus or I am still safe.”* Another participant recalled how getting tested was a way of accessing other HIV prevention services and resources: “*The reason why I go to test because I would like to know my status*,* whether I am safe or not. If I am safe*,* I will ask the doctor to help me with some condoms because at this age seriously searching for a lover*,* so that if I am negative I can protect myself.”* (man, age 19, FG#2, ID#7).

Participants discussed how positive consequences of testing included providing and receiving support from peers. A young man (age 23, FG#2, ID#3) shared how he would offer support for friends who received an HIV-positive result: “A*ccording to me*,* if my friend is HIV-positive I will not leave him alone; I will always support him and encourage him to be around people such that he does not think too much so that he can avoid committing suicide*.” In contrast, participants described negative HIV testing consequences, particularly stigma. A young woman recalled stigma if seen at a testing clinic: *“where I come from when people see you going for HIV test*,* they will just conclude that you have HIV already: that is why you are testing for it.” (woman*,* age 18*,* FG#3*,* ID#8).*

Youth revealed suicidality following learning about a positive HIV serostatus due to fear of stigma and confidentiality breaches by friends and family. One young man (age 20, FG#2, ID#8) reported: “*some friends cannot hide things: they will go to the community and spread that ‘Mr. so and so is having this sickness’*,* and this may make some people kill themselves.*” In addition, participants reported the possibility of suicide resulting from family and friend rejection upon testing HIV positive.

#### Social norms regarding gender and age

Discussions also revealed gender and age-related social norms associated with HIV testing. Participants noted women had worse experiences and consequences with HIV testing than men. One young woman (age 20, FG#1, ID#9) reported being ridiculed by men in the community for accessing HIV clinic-based testing: *“when we the ladies started lining up to go and test, men who are standing outside they started laughing.”* Due to these inequitable gender norms, a young woman perceived HIV-ST could enhance women’s privacy:

*For me there is a difference*,* because for us girls we are shy*,* so going to the hospital can make you to be shy. So it’s better for me to use that [HIV-ST] then I test myself then from there I know. For men for them they don’t care*,* even they don’t bother, they go with full force* (woman, age 22, FG#1, ID#10).

Similarly, a young man (age 24, FG#4, ID#8) commented on the extra benefits of HIV-ST for women due to community-level social norms that stigmatize women: “*with the issue of girls the way they are fearful in most things*,* I think this will favour them most because they are free to do the test at any time*,* because girls when it comes to HIV they are always more stigmatised.”*

#### Access

Issues raised regarding access to HIV testing included: limited availability of testing and antiretroviral therapy (ART); distance and language barriers; privacy and confidentiality concerns; and affordability.

#### Limited availability of HIV testing and ART

Participants perceived limited availability of HIV testing and ART in Bidi Bidi. For example, several participants discussed going to a clinic for HIV testing and being turned away due to a stockout of HIV testing supplies. This, in turn, reduced youth’s desires to seek out testing, particularly after multiple attempts. As a young woman highlighted:

*Sometimes in the hospitals*,* because those doctors sometimes they will tell you that the machines of testing this HIV is not there*,* maybe they will tell you come tomorrow*,* tomorrow you come they will tell you the same thing*,* that thing will make you to get tired to go there for the testing.* (woman, age 20, FG#1, ID#1)

Another young man highlighted a similar experience, as he felt low testing availability reflected a lack of care about his future well-being:

*They will just ask you why you are there*,* then you tell your reason that you have come for HIV testing. Immediately they will say that the test kits are now over you need to go back and come back another day*,* and when you return another [day] they will be just dodging you that the test kits are not there*,* and yet I would be wanting to know if I have the virus because I am a man and adolescent…I want to know if I am positive so that I will get the drug so that I be alive, but if the doctors tell me that the test kits are not there it means that the doctors want us to die young. (man*,* age 18*,* FG#2*,* ID#1)*

Some participants found the lack of ART availability nearby was a concerning issue. A young man explained: “*you find no drugs which can be given to you when you have tested positive of HIV and this makes it difficult*,* because there are no drugs.” (man*,* age 20*,* FG#2*,* ID#8)*.

#### Distance and language barriers

Structural barriers that constrained HIV testing access included distance and language. For many participants, clinics that offered HIV testing were a far distance from their village. One young man (man, age 23, FG#2, ID#3) expressed: “*The health centre that we are using is in Bolomoni*,* which is like four kilometres*,* so it’s very far for us.”* Conversely, participants with reduced travel distance to clinics reported improved testing access. Additionally, youth discussed language barriers in healthcare interactions that reduced HIV testing engagement. As a young man reported: “*Some doctors only know English and the language of the tribes they come from. Now*,* when you visit such a doctor*,* you may not have the language that you can use to freely communicate with him or her.” (man*,* age 19*,* FG#2*,* ID#2)*.

Some participants perceived HIV-ST as a way to improve access, as highlighted by a young woman: *“For me it is very nice because if they offer me that thing [HIV-ST] I will go in my room and test myself and I see if I am positive or negative and if I am positive I see the decision to take to go to the health centre and take ARVs.” (age 22*,* FG#3*,* ID#4).*

#### Clinic privacy and confidentiality concerns

Privacy and confidentiality concerns were identified as barriers to accessing clinic-based HIV testing. For example, a young man discussed the clinic location raised privacy concerns:

*The quality of service is okay but the problem is (X) health centre is just located opposite the school*,* so during lunch break some pupils run to the health centre to take water. Now if you happen to reach the health centre to test*,* you will find very many pupils and some of them even know you so you will just develop fear because you will think that they will get to know your status. (man*,* age 22*,* FG#4*,* ID#7)*

Additionally, participants shared concerns regarding confidentiality breaches by healthcare workers and translators. As a young woman explained:

*There is lack of confidentiality from health workers*,* some of them don’t keep secrets instead of … maybe giving you advice on how to take the drugs*,* they will speak well here but later on they will go and tell people that “you see this one here bragging here for nothing she is just rotten.” (woman*,* age 20*,* FG#1*,* ID#9)*.

Others discussed receiving poor treatment from healthcare providers: “*if you go to the health centre for testing some doctors*,* after knowing that you are having HIV positive [test] they will even disrespect you*,* even they will quarrel at you. Before they give the advice*,* they must say something bad for you.” (woman*,* age 16*,* FG#1*,* ID#6)* Other access barriers included judgment and stigma in healthcare settings when seeking HIV testing. Participants discussed how this could be exacerbated by age differences between youth and healthcare providers, as a young man explained (age 18, FG#2, ID#1): “*young people fear to go for testing because they look at themselves as young and the doctors are older so how to start asking the doctors to test them for HIV is very hard.”*

Participants discussed HIV-ST as a strategy with the potential to mitigate these privacy and confidentiality concerns. For example, a young woman (age 17, FG#1, ID#5) explained how: *“It’s good because it’s confidential*,* because if you find yourself positive you can keep it to yourself and it will prevent other people from talking negatively.”* Conversely, some participants wanted healthcare provider support when testing and worried about how to manage a positive HIV-ST result: *“For me I will not welcome it [HIV-ST] because when I test myself I will be there with my problems and I will just bury myself there. According to me*,* someone should go to the health clinic to get advice and counselling and drugs immediately”(woman*,* age 18*,* FG#3*,* ID#1).*

#### Affordability

Participants reported differences in HIV testing costs across venues. Testing at private health clinics was described as unaffordable. As one young man (man, age 23, FG#2, ID#3) reported: *“for the clinic we are charged three thousand Ugandan shillings for testing*,* which is not cheap at all.”* Alternatively, testing is free at public health centres. Others noted HIV-ST could eliminate the cost of traveling to health centres/clinics: *“the self-test kit will help reduce on the distance since you can just do it from home; I will be very willing to use it because it will not cost me anything” (man*,* age 21*,* FG#4*,* ID#4).*

#### Effective Use

Focus group discussions illustrate effectiveness regarding engaging in HIV testing is shaped by: HIV serostatus knowledge self-efficacy and in/equitable partner dynamics.

#### HIV serostatus knowledge self-efficacy

Several participant discussions reflect the concept that HIV-ST can be empowering due to acquiring knowledge of one’s HIV status and being able to personally care for one’s health. For example, one young man stated how knowing his HIV serostatus could provide the opportunity to take care of his own health: *“if I test myself and I am positive I will not panic*,* I will just start medication immediately and I will also protect myself from getting new infections” (man*,* age 18*,* FG#4*,* ID#5).*

In another example, a young woman mentioned that knowledge of her status would improve her life when discussing if she would accept an HIV-ST kit from a peer educator: *“I will go [for HIV-ST] wholeheartedly because I know that they love me and they want me to know my status so I can know myself and live a better life”(woman*,* age 23*,* FG#3*,* ID#9).*

#### In/equitable partner dynamics

There was complexity regarding partner dynamics. For instance, partner testing was seen as beneficial within trusting relationships: *“if I have my lover and I love her so much and I trust her we can use this test kit to test ourselves together so that we both can know each other’s result” (man*,* age 19*,* FG#2*,* ID#2).* Partner testing could also reduce uncertainty about a partner’s HIV serostatus: *“to me it is good to get tested with your boyfriend: sometimes you may not know someone’s status but when you test together you can get to know each other’s status” (woman*,* age 21*,* FG#3*,* ID#2).* In such cases, partners could support one another in case one or both persons test HIV-positive.

Participants also raised concerns about the potential harms of partner testing, including women being blamed for bringing HIV into the relationship and separation/divorce. A young woman (age 20, FG#1, ID#1) warned of the potential blame placed on women: *“if someone has a bad heart [and thinks] that maybe you are the one who give me this disease*,* then from there you end up fighting*,* separating yourselves.”* Moreover, women who test HIV-positive may experience heightened risks of gender-based violence (GBV). One young man (man, age 21, FG#4, ID#3) explained: *“to me I don’t see it [partner testing] as something good because you can test together and find that one person is healthy and the other is positive*,* in this case if it is the woman who is positive she will take this message to their home so it may bring violence at home.”* This fear of GBV was reiterated by a young woman (age 21, FG#3, ID#30): *“others will be having that fear that he will beat you and even want to kill you if you are positive.”*

## Discussion

Refugee youth in Bidi Bidi reported complex factors that shape motivation for, access to, and effective use of HIV testing. Participants indicated an interest in HIV-ST to mitigate testing barriers, however, underlying misinformation and inequitable social and gender norms still need attention to optimize HIV testing.

Our findings corroborate prior research on *motivation barriers* such as intersecting stigma among urban refugee youth, including social and healthcare marginalization at the nexus of youth, women, and refugee social categories [13, 36]. We found HIV misinformation spanned HIV treatment, testing, and acquisition risks. While prior research documented associations between lower education and reduced lifetime HIV testing odds among urban refugee youth in Kampala [4], our findings signal broader knowledge gaps regarding the general concept of *prevention literacy* among refugee youth that includes “understanding relatively complex technical information about a growing range of prevention methods but also on the question of access as a fundamental human right.”(p. 4) [37]. As evidenced through *motivation* factors such as low testing knowledge and risk perception, there are underlying myths and beliefs regarding HIV testing (e.g., if one’s friend is HIV negative their friends would also be HIV negative) which reflect low prevention literacy and can decrease HIV testing motivation [37]. Refugee youth participants also provided examples which reveal they were unaware of the human right to HIV testing access—a tenet of prevention literacy [37]. For instance, participants explained how girls and young women were ridiculed and shamed for seeking clinic-based HIV testing.

Our findings build on research regarding documented HIV and STI testing *access barriers* among youth in LMIC, including limited availability of SRH supplies, insufficient staffing, unsupportive healthcare provider attitudes, and lack of confidential, youth-friendly care [38]. We also build on prior research on stockouts of SRH supplies in humanitarian settings [8, 19, 20] to reveal gaps in HIV testing supplies and how this affects refugee youth who can feel frustrated and discouraged—and at times devalued—with continued attempts to access testing. Findings also align with prior research on logistical testing barriers (language, literacy, travel distance) [14, 39, 40], which may be exacerbated in large refugee settlements with limited transport options such as Bidi Bidi.

Our research expands knowledge of *effective use* regarding HIV testing through noting the salience of self-efficacy via HIV serostatus knowledge, and considerations of complex relationship power dynamics. Our finding that HIV serostatus knowledge would empower youth to engage in HIV treatment and care reflects the importance of treatment literacy and acknowledges processes of empowerment and consciousness-raising when people can both learn and apply this knowledge into action [37]. For some young people, such as South African non-refugees, partner testing can offer support and encouragement to begin HIV treatment following a positive diagnosis [21]. While this was indeed noted in participant discussions, participants also reinforced concerns brought up in prior research with urban refugee youth that inequitable power dynamics elevate young women’s risk of GBV when testing HIV-positive in a partner’s presence [13, 15].

The HIV prevention cascade [16] offers a framework to leverage findings to inform intervention development. For instance, strategies to address HIV testing motivation in Bidi Bidi can include creative information campaigns, such as arts-based [41], peer-based [42], and mass media [43] models. To address access, youth-friendly, non-stigmatizing healthcare service delivery [44, 45] and gender-transformative programming [46, 47] is needed, alongside minimizing stockouts that could be aided with HIV-ST provision [8]. Strategies for effective HIV testing use can utilize treatment literacy approaches that build agency and consciousness-raising via popular education, community dialogue, and social support networks, as well as fostering technical (e.g., information) and transformative (e.g., agentic environments) communication [48, 49]. As refugee youth may experience complex, intergenerational violence trajectories [50], integrating GBV and HIV services is key [51], as is considering contextually, age, and gender-tailored approaches for incorporating GBV screening and prevention into refugee youth HIV prevention and care services [52].

Limitations include the focus group format, as participants may have felt uncomfortable sharing HIV testing experiences among peers. We did not explore sexual and gender diversity due to the political climate of criminalization, thus findings do not reflect LGBTQ refugee experiences. As URDMC recruited participants, the sample may be more aware of HIV and prevention services. As such, this convenience sample of youth might not share the same experiences of HIV testing drivers of motivation, access, and effective use as other youth in Bidi Bidi—as there is a large and heterogeneous refugee youth population. Despite these limitations, our findings offer unique insight into refugee youth HIV testing, including HIV-ST, in one of the world’s largest refugee settlements with implications for interventions guided by the HIV prevention cascade [16] (Fig. 1).

## Conclusions

Our findings signal the applicability of the HIV prevention cascade framework [16] for identifying motivation, access, and effective use domains relevant to HIV testing engagement among refugee youth. Findings can inform tailored intervention strategies that facilitate HIV prevention *and* treatment literacy [37] and adaptation and implementation of evidence-based strategies to reduce gender inequity, GBV, and intersecting stigmas [53] to a refugee settlement context. For instance, we designed and implemented the *Todurujo na Kadurok* (loosely translated to ‘Empowering youth’ in Bari) HIV-ST study with refugee youth in Bidi Bidi building on these findings to address factors related to motivation, access, and effective use in order to increase HIV testing uptake [29]. Efforts such as these that address the complexities of the HIV prevention cascade hold the potential to address larger social and structural inequities and in turn optimize HIV testing engagement with refugee youth in LMIC humanitarian settings, including and extending beyond Bidi Bidi.

## Electronic supplementary material

Below is the link to the electronic supplementary material.

Supplementary Material 1

## Data Availability

The datasets used and/or analysed during the current study available from the corresponding author on reasonable request and upon obtaining ethics board approval.
