# Supplementary material for: Contextualizing HIV testing experiences within the HIV prevention cascade: qualitative insights from refugee youth in Bidi Bidi refugee settlement, Uganda
Source: BMC Public Health. 2024 Sep 27;24:2599. doi: 10.1186/s12889-024-20135-2 (PMC11429596; doi:10.1186/s12889-024-20135-2)
Supplement: Supplementary file 1 — Supplementary Material 1 [file 12889_2024_20135_MOESM1_ESM.pdf]

INTERVIEW GUIDE EXAMPLE QUESTIONS

1. Please tell me about the HIV testing that is available for refugee young people in Bidi Bidi

    a. Can you tell me about what options that are there for HIV testing in your community?
    b. What do people in your community think about these HIV testing options? (prompt: location, transportation, staff, quality, confidentiality, cost, comfort)
    c. How is this different for boys and girls?

    To note: if focus group participants do not know about HIV testing options in Bidi Bidi, ask them whether they know any in Yumbe? If they do not know any, then you can tell them that HIV testing is available at clinics and ask "What do you think young people in your community would think about going to a clinic in Bidi Bidi for HIV testing? What about in Yumbe?" Then you can continue to ask the rest of the questions below.

2. What helps young people in your community decide to get an HIV test?

3. What are some challenges young people in your community face for testing? (e.g. stigma, location, hours)

4. What does it mean to be a young man/woman *(use the gender that relates to the focus group: for young men, ask "young men")* in your community?

    Pause and allow them to answer, then probe further by asking:
    a. What do people in your community think of a young man/young woman (depending on group) who decides to get an HIV test?
    b. What kinds of challenges or concerns might young men/young women (depending on group) face that are unique to young men?

5. How are people living with HIV treated in your community?

    Pause and allow them to answer, then probe further by asking:
    a. What might be concerns that a young person would have if they received a positive test for HIV? (e.g. stigma, violence)
    b. How is this different for boys and girls?
    c. What do you suggest could be solutions in your community to support people living with HIV?
    d. How might we reduce stigma toward HIV in your community?

Aim of the questions in this next section is to find out what young people in Bidi Bidi think about HIV self-testing.

6. New HIV tests have been developed so that people can test themselves without the need to go to a clinic. You can do this new test yourself at your own home by swabbing the inside of your cheek. The test results are available within 20 minutes, and there are instructions provided on how to read the result. If it's an HIV positive result, it's very important to go to the clinic to confirm the result.
    a. How would you/your peers feel about being offered self-testing for HIV (an oral HIV test that you could conduct yourself at home)?

       Pause and allow them to answer, then probe further by asking:

    b. What do you think about this idea of testing yourself for HIV at home?
    c. What do you think your friends would think of testing themselves for HIV at home?
    d. How would this be different for boys and girls?
    e. What might **motivate** young people to use an HIV self-testing kit?
    f. What might be some **challenges** or concerns you or your peers would have with HIV self-testing?
       Allow the participants to answer freely but try to discuss all of the following challenges:

       - *where to dispose of the kit,*
       - *interpreting the result,*
       - *lack of privacy,*
       - *lack of support,*
       - *location for receiving the test and*
       - *location of using the test.*

       For each challenge described ask "What would you suggest to do to address this challenge?"

7. I would now like to show you the self-testing kit we would like to use in Bidi Bidi. I will explain the instructions to you and show you the written instructions and diagrams.
    a. What do you think about the instructions? *(probe: easy, clear)*
    b. What other information do you think you/your peers would like to know before testing?

8. Now that you have learnt about HIV self-testing, how willing would young people like you be to use an HIV self-test?

Pause and allow them to answer, then probe further by asking:

a. How would you feel about the following persons offering you an HIV self-test kit *(pause and allow them to answer after each option i-iv)*:
    i. a peer educator
    ii. a friend
    iii. a family member
    iv. a sexual partner
b. What are your thoughts about testing using an HIV self-test together at the same time as your sexual partner? what are some pros? Some cons?
